# Evaluation of water quality and stability in the drinking water distribution network in the Azogues city, Ecuador

**DOI:** 10.1016/j.dib.2018.03.007

**Published:** 2018-03-09

**Authors:** Fernando García-Ávila, Lía Ramos- Fernández, Damián Pauta, Diego Quezada

**Affiliations:** a Universidad Nacional Agraria La Molina, Lima, Perú; b Facultad de Ciencias Químicas, Universidad de Cuenca, Ecuador

## Abstract

This document presents the physical-chemical parameters with the objective of evaluating and analyzing the drinking water quality in the Azogues city applying the water quality index (WQI) and to research the water stability in the distribution network using corrosion indexes. Thirty samples were collected monthly for six months throughout the drinking water distribution network; turbidity, temperature, electric conductivity, pH, total dissolved solids, total hardness, calcium, magnesium, alkalinity, chlorides, nitrates, sulfates and phosphates were determined; the physical-chemical parameters were measured using standard methods. The processed data revealed that the average values ​​of LSI, RSI and PSI were 0.5 (±0.34), 6.76 (±0.6), 6.50 (±0.99) respectively. The WQI calculation indicated that 100% of the samples are considered excellent quality water. According to the Langelier, Ryznar and Pukorius indexes showed that drinking water in Azogues is corrosive. The quality of drinking water according to the WQI is in a good and excellent category.

**Specifications table**

**Table:** 

Subject area	Environmental Engineering
More specific subject area	Water treatment
Type of data	Table and figure
How data was acquired	In 30 points of the drinking water network for six months, 176 water samples were collected, stored and transferred to the lab using standard methods and the drinking water quality; turbidity, pH, temperature, total dissolved solids, total hardness, calcium hardness, alkalinity, nitrate, phosphate, chloride, sulfate and free chlorine were measured. Alkalinity, total hardness and calcium hardness were measured by titration method; the hydrogen ion concentration (pH), temperature and total dissolved solids were analyzed with the HACH Multiparameter HQ 40d. Turbidity were measured with turbidimeter (model P2100 HACH); nitrate, phosphate, chloride, and sulfate were determined with HACH DR 2500 spectrophotometer, free chlorine were measured with HACH DR890 and compared with internal standards.
Data format	Raw, analyzed
Experimental factors	The mentioned parameters above, in abstract section, were analyzed according to the standards for water and wastewater treatment handbook.
Experimental features	The levels of physical and chemical parameters drinking water were determined.
Data source location	Azogues, Ecuador 2°44'22" S, 78°50'54" O
Data accessibility	Data are available in the article.

**Value of the data**

• The dates presented are used to calculate the water quality indexes, as well as the corrosion and scaling indexes, emphasizing the importance of continuous monitoring of water quality. Determine the corrosion potential and quality of drinking water in all distribution systems is important to avoid adverse effects on health and economic losses due to deterioration of the infrastructure.
• The water quality indexes (WQI) serve to provide a clear picture of the quality of water distributed and used for human consumption, therefore, these data could be useful for communities, or cities that have similar drinking water quality.
• The pipes, fittings and valves in the distribution networks deteriorate due to corrosive water and cause some health, aesthetic and economic problems. Then, the determination of the corrosion and incrustation potential of drinking water allows decision making and adoption of guidelines for the management of water quality by those who are related to engineering and water quality management.
• Sharing such data can allow a much earlier rectification of the problem of corrosion and scaling.

## 1 Data

The data presented in this article deals with the quality of drinking water distributed in the Azogues city, Ecuador. Data include in this document, indicate about the situation of drinking water saturation, three stability indexes were determined: Langelier, Ryznar, and Pockorius, were calculated using special equations that are summarized in Table 1. Other data parameters such as turbidity (Tur), pH, total dissolved solids (TDS), electrical conductivity (EC), total hardness (TH), calcium (Ca^**2+**^), magnesium (Mg^**2+**^), alkalinity (Alk), sulfate (SO_**4**_^**2−**^), chloride (Cl^**−**^), nitrate (NO_**3**_^**−**^), phosphate (PO_**4**_^**3−**^), free chlorine (Cl_2_). The physical and physical characteristics of drinking water are shown in Table 2, Table 3. The corrosion indexes values obtained are presented in Table 4.

**Table 1:** Equations and classifications of Langelier, Ryznar and Pockorius indexes [2], [3], [4].

**Index**	**Equation**	**Description**	**Value**	**Water condition**
Langelier	LSI = pH-pHs	LSI = Langelier Saturation Index	LSI > 0	Tend to precipitate
LSI		pH = pH measured in situ.		
				
		pHs = pH at saturation		
		
		pHs = 9.3 + A + B - C - D	LSI = 0	Equilibrium
		
		$A=\frac{(\log10\mathrm{TDS}–\;1)}{10}$		
		B = [−13.12log_10_(273 °C+T)]+34.55 C = log_10_[${\mathrm{Ca}}^{+2}$mg/L as ${\mathrm{CaCO}}_{3}$]−0.4	LSI < 0	Tend to corrosion
		
		D = log10 [Alk. mg/L as ${\mathrm{CaCO}}_{3}$]		
		TDS = Total Dissolved Solids mg/L		
Ryznar	RSI = 2 pHs-pH	RSI = Ryznar Stability Index	RSI < 5.5	Highly scale-forming
				
RSI		pHs = pH at saturation		
		pH = pH measured in situ	5.5 < RSI < 6.2	Relatively scale-forming
		
			6.2 < RSI < 6.8	Balanced
	
			6.8 < RSI < 8	Low corrosion
			RSI > 8	High corrosion
Puckorius	PSI = 2pHs−pHeq		PSI < 5.5	Tend to precipitate
				
PSI		pHs = pH at saturation	5.5<PSI<6.5	Optimal range
		pHeq = 1.456 Log(Alk) + 4.54	PSI > 6.5	Tend to corrosion

**Table 2:** Values of the physico-chemical parameters analyzed.

**Number Sample**	**Turbidity NTU**	**pH**	**Temp. °C**	**TDS mg/L**	**EC µS/cm**	**Total hardness mg/L CaCO**_**3**_	**Ca**^**2+**^**mg/L CaCO**_**3**_	**Ca**^**2+**^**mg/L**
S1	0.56	7.42	17.52	81.17	129.96	68.33	51.00	20.40
S2	0.55	7.38	19.13	82.17	131.83	70.00	52.17	20.87
S3	0.51	7.20	16.68	63.67	102.49	69.33	52.17	20.87
S4	0.61	7.17	20.09	80.67	129.49	78.00	57.83	23.13
S5	0.59	7.46	18.46	70.40	111.89	72.00	56.00	22.40
S6	0.57	7.26	17.32	73.60	117.62	75.60	58.00	23.20
S7	0.51	7.22	18.25	75.67	119.47	70.67	53.67	21.47
S8	0.50	7.21	16.78	75.60	120.82	74.00	56.00	22.40
S9	0.59	7.20	18.33	64.83	103.27	69.00	52.67	21.07
S10	0.47	7.20	16.88	66.17	106.09	69.83	52.00	20.80
S11	0.45	7.17	15.93	71.17	112.96	71.17	53.00	21.20
S12	0.49	7.22	15.30	66.83	106.33	71.50	52.67	21.07
S13	0.44	7.16	14.85	58.67	94.61	56.33	42.00	16.80
S14	0.54	7.26	18.13	72.00	115.01	70.83	55.00	22.00
S15	0.55	7.35	16.95	72.50	115.37	73.17	54.67	21.87
S16	0.55	7.16	19.75	74.83	119.11	72.00	54.33	21.73
S17	0.53	7.20	17.20	71.00	112.89	71.10	53.50	21.40
S18	0.54	7.22	18.10	70.17	111.16	72.42	54.75	21.90
S19	0.50	7.23	17.32	69.00	110.58	74.83	55.67	22.27
S20	0.50	7.18	18.57	71.33	112.57	70.83	53.50	21.40
S21	0.52	7.22	17.62	55.67	88.36	55.50	42.17	16.87
S22	0.50	7.19	17.53	64.17	103.92	65.17	50.83	20.33
S23	0.48	7.27	17.03	66.50	106.29	69.83	54.00	21.60
S24	0.48	7.24	19.08	68.83	109.29	68.00	52.67	21.07
S25	0.47	7.22	16.75	63.67	99.96	65.67	50.50	20.20
S26	0.47	7.26	18.58	67.50	106.85	65.33	49.33	19.73
S27	0.49	7.26	16.67	67.17	107.07	67.67	51.33	20.53
S28	0.47	7.28	15.72	66.83	106.08	67.33	50.67	20.27
S29	0.53	7.20	20.08	73.50	117.72	67.17	52.00	20.80
S30	0.50	7.27	17.97	55.67	88.68	58.50	44.17	17.67
Mean	0.51	7.24	17.6	69.29	110.47	68.94	52.19	20.88
Min	0.39	7.16	14.8	55.67	88.36	55.50	42.00	16.80
Max	0.61	7.46	20.1	82.17	131.83	78.00	58.00	23.20
S.D.	0.05	0.07	1.3	6.55	10.52	5.12	3.84	1.54

**Table 3:** Values of the physico-chemical parameters analyzed.

**Number sample**	**Mg**^**2+**^**mg/L CaCO**_**3**_	**Mg**^**2+**^**mg/L**	**Free chlorine mg/L**	**SO**_**4**_^**2−**^**mg/L**	**Alkalinity mg/L CaCO**_**3**_	**Cl**^**−**^**mg/L**	**NO**_**3**_^**−**^**mg/L**	**PO**_**4**_^**3−**^**mg/L**
S1	17.33	4.16	0.47	22.33	46.83	5.30	0.55	0.07
S2	17.83	4.28	0.59	21.00	50.00	5.58	0.58	0.07
S3	17.17	4.12	0.76	19.17	46.33	5.02	0.48	0.07
S4	20.17	4.84	0.82	23.00	48.67	5.22	0.37	0.07
S5	16.00	3.84	0.53	20.20	48.00	5.22	0.38	0.07
S6	17.60	4.22	0.71	19.80	54.80	5.24	0.44	0.07
S7	17.00	4.08	0.45	18.67	52.50	5.12	0.50	0.11
S8	18.00	4.32	0.75	18.90	56.60	5.88	0.50	0.09
S9	16.33	3.92	0.56	17.33	50.00	5.47	0.38	0.07
S10	17.83	4.28	0.69	18.00	51.33	5.65	0.42	0.07
S11	18.17	4.36	0.71	15.83	51.17	5.55	0.52	0.08
S12	18.83	4.52	0.90	16.50	53.33	4.88	0.43	0.06
S13	14.33	3.44	0.55	12.17	40.33	7.27	0.67	0.06
S14	15.83	3.80	0.62	20.67	47.67	5.25	0.58	0.06
S15	18.50	4.44	0.64	20.50	48.83	5.63	0.50	0.08
S16	17.67	4.24	0.75	21.83	45.83	5.28	0.35	0.24
S17	17.60	4.22	0.86	20.00	46.60	5.72	0.44	0.08
S18	17.67	4.24	0.86	19.33	50.00	5.55	0.47	0.07
S19	19.17	4.60	0.66	19.83	47.50	5.35	0.63	0.08
S20	17.33	4.16	0.80	18.67	46.68	5.75	0.53	0.08
S21	13.33	3.20	0.58	11.00	40.82	6.52	0.53	0.07
S22	14.33	3.44	0.84	17.83	46.33	5.85	0.37	0.07
S23	15.83	3.80	0.85	20.17	48.50	5.25	0.35	0.07
S24	15.33	3.68	0.78	18.00	46.50	5.90	0.50	0.07
S25	15.17	3.64	0.86	17.00	47.17	5.40	0.37	0.08
S26	16.00	3.84	0.82	16.17	48.83	5.88	0.38	0.09
S27	16.33	3.92	0.81	17.00	48.17	6.08	0.43	0.08
S28	16.67	4.00	0.86	16.67	50.67	6.15	0.65	0.10
S29	15.17	3.64	0.77	17.00	49.33	5.67	0.60	0.08
S30	14.33	3.44	0.48	10.17	40.33	6.43	0.47	0.08
Mean	16.75	4.02	0.71	18.12	48.25	5.64	0.48	0.08
Min	13.33	3.20	0.5	10.17	40.33	4.88	0.35	0.06
Max	20.17	4.84	0.9	23.00	56.60	7.27	0.67	0.24
S.D.	1.60	0.38	0.1	3.02	3.70	0.50	0.09	0.03

**Table 4:** Corrosion and scaling indexes in drinking water of Azogues city.

**Number sample**	**Langelier index LSI**	**Ryznar index RSI**	**Puckorius index PSI**
S1	−1.25	9.92	10.38
S2	−1.22	9.81	10.19
S3	−1.42	10.04	10.21
S4	−1.38	9.92	10.09
S5	−1.15	9.76	10.25
S6	−1.28	9.82	10.00
S7	−1.35	9.93	10.11
S8	−1.34	9.89	10.00
S9	−1.39	9.98	10.16
S10	−1.42	10.04	10.21
S11	−1.46	10.09	10.23
S12	−1.41	10.03	10.20
S13	−1.68	10.52	10.79
S14	−1.35	9.97	10.26
S15	−1.26	9.88	10.23
S16	−1.44	10.03	10.23
S17	−1.44	10.08	10.32
S18	−1.36	9.94	10.15
S19	−1.39	10.01	10.26
S20	−1.43	10.04	10.25
S21	−1.55	10.32	10.65
S22	−1.47	10.12	10.34
S23	−1.35	9.98	10.26
S24	−1.37	9.98	10.24
S25	−1.44	10.10	10.34
S26	−1.36	9.99	10.25
S27	−1.39	10.04	10.31
S28	−1.37	10.03	10.29
S29	−1.37	9.93	10.13
S30	−1.48	10.23	10.61

**Fig. 1:**
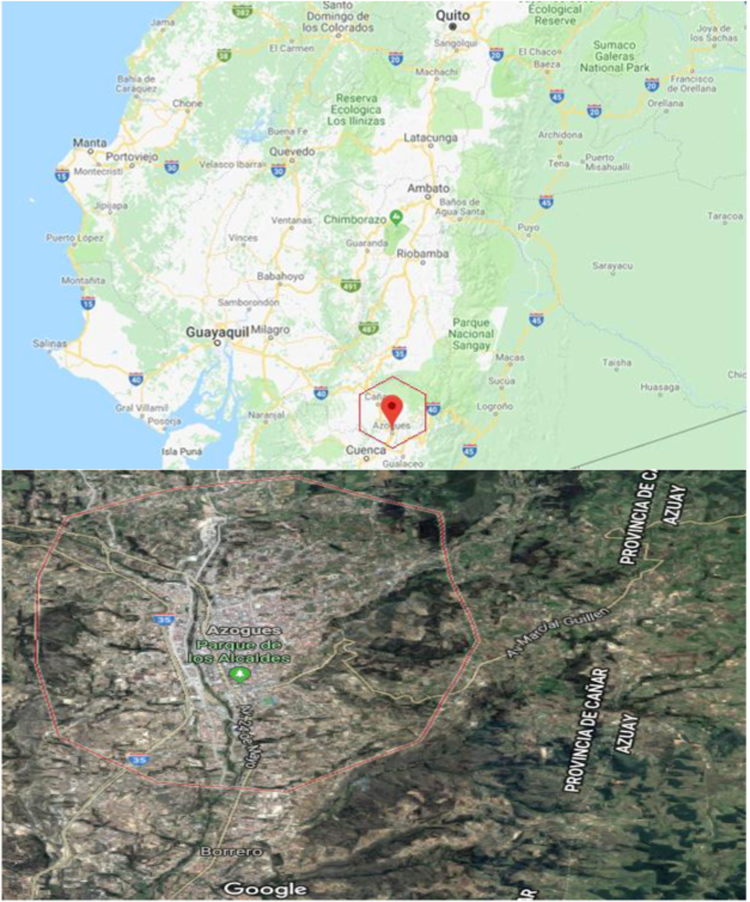
Map and location of Azogues city.

Also in this document the drinking water quality indexes (WQI) are presented, to evaluate the water quality through WQI, the recommended standards by the World Health Organization (WHO) 2011 were considered [1] the relative weight (Wi) was assigned for water quality parameters according to their relative importance to water quality for consumption purposes (Table 5). The calculation obtained from WQI for drinking water samples is presented in Table 7. The classification of drinking water quality based on WQI values is shown in Table 8. Azogues city where the study was realized is shown in Fig. 1.

**Table 5:** Relative weight of chemical of physico-chemical parameters [2], [8], [9], [10], [11].

**Chemical parameter**	**Unit**	**Factor weight (wi)**	**WHO standard (si)**	**Relative weights (Wi)**
Turbidity	NTU	5	0.5	0.12
pH		4	8.5	0.10
TDS	mg/L	3	500	0.07
Total Hardness	mg/L	2	200	0.05
Calcium Ca^2 +^	mg/L	3	75	0.07
Magnesium Mg^2+^	mg/L	2	50	0.05
Sulfate	mg/L	4	250	0.10
Alkalinity	mg/L	3	200	0.07
Chloride	mg/L	3	250	0.07
Nitrate	mg/L	5	50	0.12
Phosphate	mg/L	2	0.5	0.05
Free Chlorine	mg/L	5	1	0.12

**Fig. 2:**
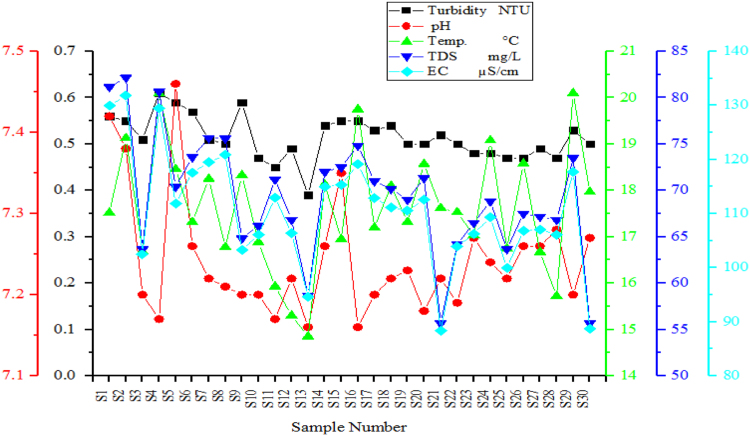
Variation of turbidity, pH, Temperature, Dissolved Total Solids and Electric Conductivity in the sampling points of the drinking water network.

**Fig. 3:**
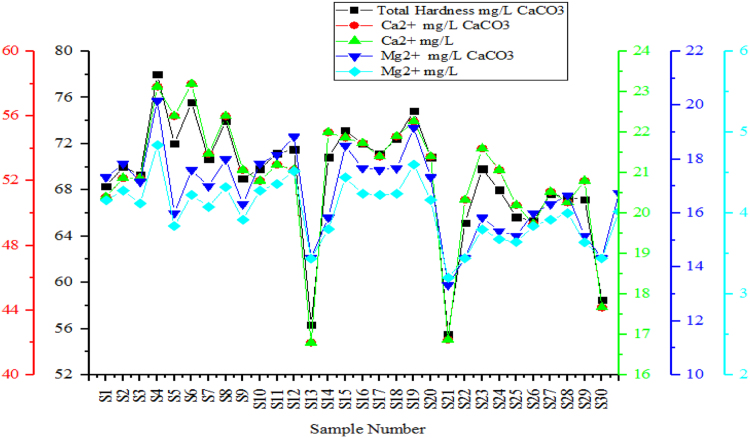
Variation of Total Hardness, Calcium and Magnesium in the sampling points.

**Fig. 4:**
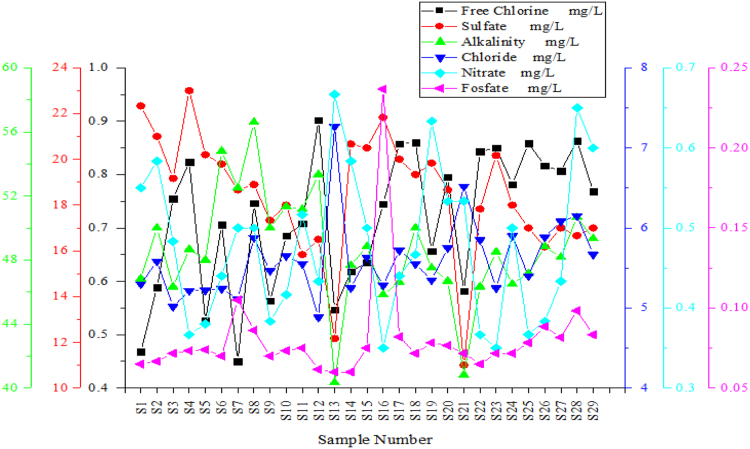
Variation of Free Chlorine, Alkalinity, Sulphates, Chlorides, Nitrates, Phosphates in the sampling points of the drinking water network.

**Fig. 5:**
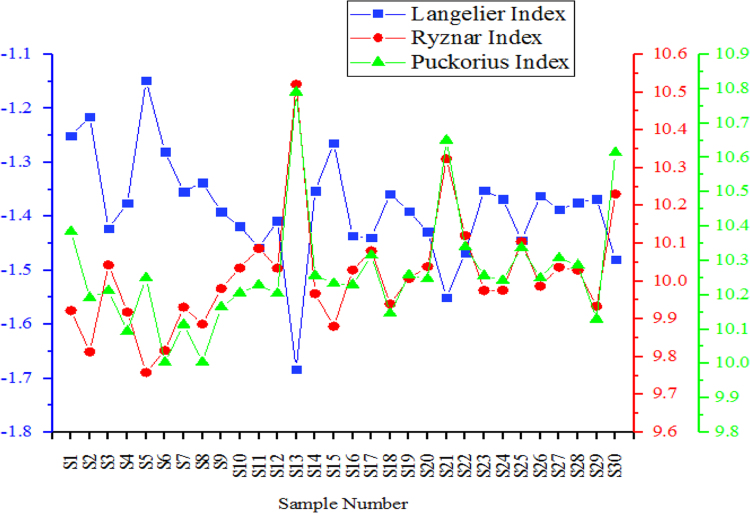
Trend of Langelier, Ryznar and Pockorius indexes.

**Fig. 6:**
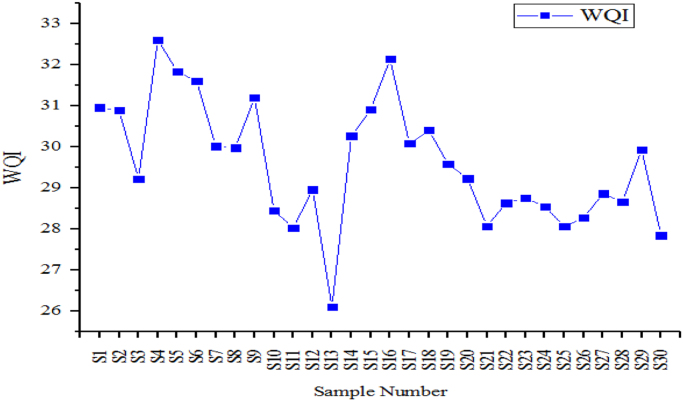
Trend of water quality indices in the 30 sampling points.

From Fig. 2, Fig. 3, Fig. 4 the variation of the physical-chemical parameters in the 30 sampling points is shown. Fig. 5 shows the variation of the corrosion indexes in the 30 sampling points. Fig. 6 show the Water Quality Indexes at the 30 sampling points.

## 2 Experimental design, materials and methods

### 2.1 Study area description

The Azogues city is located south of the Republic of Ecuador, its geographic coordinates are: latitude 2° 44'22 "S, longitude: 78° 50'54" W, they cover an area of approximately 1200 km^2^, the average altitude of the city is 2518 m above sea level, the average temperature is 17 °C.

**Fig. 7:**
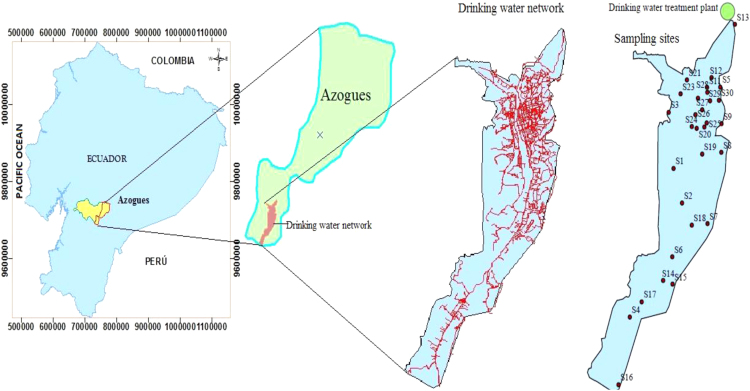
Location of the water sampling sites in the Azogues city drinking water network.

Fig. 7 shows the location of the drinking water network.

### 2.2 Collection of samples and analytical procedures

Monthly samples were collected at 30 points of the drinking water network for six months, 180 samples in total were collected, stored and transferred to the laboratory using standard methods and drinking water quality; turbidity, pH, temperature, total dissolved solids, total hardness, calcium hardness, alkalinity, nitrate, phosphate, chloride, sulfate and free chlorine were measured. Fig. 7 shows the study area and the sampling locations, the samples were collected in polyethylene bottles (1 L) and transported immediately at 4 °C to the central laboratory of the drinking water company. The alkalinity, total hardness and hardness of calcium were measured by the titration method; concentration of hydrogen ion (pH), temperature and total dissolved solids were analyzed with HACH Multiparameter HQ 40d, turbidity was measured using turbidimeter (model P2100 HACH); nitrate, phosphate, chloride and sulfate were determined with the HACH DR 2500 spectrophotometer, free chlorine was measured with HACH DR 890. All the water samples were analyzed according to the standard methods for the analysis of drinking water [5], [6], [7]. The data obtained after the laboratory analysis is presented in Table 2, Table 3.

### 2.3 Drinking water stability indexes calculation

Table 1 presents the equations and criteria to calculate and categorize the water stability indexes. The Langelier saturation index, Ryznar saturation index, and the Puckorius scale index were calculated and classified into three categories: scale, stabilized and corrosive [2], [3], [4]. The results are presented in Table 4.

### 2.4 Water quality index calculation

For calculation of WQI, the following four steps have been taken into account [8], [9], [10], [11]. In the first step, each of the analyzed parameters has been assigned a weight (wi) according to its relative importance in the overall quality of water for drinking purposes (Table 5). In the second step, the relative weight (Wi) is calculated as per the established method as follows.

$$W_{i}=\frac{w_{i}}{\sum_{i=1}^{n}w_{i}}$$

Where ‘Wi’ is the relative weight, ‘wi’ is the weight of each parameter and ‘n’ is the number of parameters. In the third step a quality rating scale (qi) for each parameter is calculated by following equation (Table 6);

$$q_{i}=\frac{C_{i}}{s_{i}}100$$

Where ‘Ci‘ is the concentration of each chemical parameter in each water sample and ‘si’ is the standard value for each chemical parameter according to the Guide lines of WHO. In the fourth step the sub index (Sli) of each chemical parameter is estimated by using the equation;

$${\mathrm{SI}}_{i}=W_{i}xq_{i}$$

**Table 6:** Data of quality rating (qi).

**Number sample**	**Tur.**	**pH**	**TDS**	**TH**	**Ca**^2 +^	**Mg**^2 +^	**SO**_**4**_^**2−**^	**Alk.**	**Cl**^**−**^	**NO**_**3**_^**−**^	**PO**_**4**_^**3−**^	**Cl**_**2**_
S1	112	87.29	16.23	34.17	27.20	8.32	8.93	23.42	2.12	1.10	13.00	0.23
S2	110	86.82	16.43	35.00	27.83	8.56	8.40	25.00	2.23	1.17	13.33	0.29
S3	102	84.71	12.73	34.67	27.83	8.24	7.67	23.17	2.01	0.97	14.33	0.38
S4	122	84.35	16.13	39.00	30.84	9.68	9.20	24.33	2.09	0.73	14.67	0.41
S5	118	87.76	14.08	36.00	29.87	7.68	8.08	24.00	2.09	0.76	14.80	0.26
S6	114	85.41	14.72	37.80	30.93	8.45	7.92	27.40	2.10	0.88	14.00	0.35
S7	102	84.94	15.13	35.34	28.63	8.16	7.47	26.25	2.05	1.00	21.00	0.23
S8	100	84.82	15.12	37.00	29.87	8.64	7.56	28.30	2.35	1.00	17.20	0.37
S9	118	84.71	12.97	34.50	28.09	7.84	6.93	25.00	2.19	0.77	14.00	0.28
S10	94	84.71	13.23	34.92	27.73	8.56	7.20	25.67	2.26	0.83	14.67	0.34
S11	90	84.35	14.23	35.59	28.27	8.72	6.33	25.58	2.22	1.03	15.00	0.35
S12	98	84.94	13.37	35.75	28.09	9.04	6.60	26.67	1.95	0.87	12.33	0.45
S13	88	84.24	11.73	28.17	22.40	6.88	4.87	20.17	2.91	1.33	12.00	0.27
S14	108	85.41	14.40	35.42	29.33	7.60	8.27	23.83	2.10	1.17	12.00	0.31
S15	110	86.47	14.50	36.59	29.16	8.88	8.20	24.42	2.25	1.00	15.00	0.32
S16	110	84.24	14.97	36.00	28.97	8.48	8.73	22.92	2.11	0.70	47.33	0.37
S17	106	84.71	14.20	35.55	28.53	8.45	8.00	23.30	2.29	0.88	16.40	0.43
S18	108	84.94	14.03	36.21	29.20	8.48	7.73	25.00	2.22	0.93	14.33	0.43
S19	100	85.06	13.80	37.42	29.69	9.20	7.93	23.75	2.14	1.27	15.67	0.33
S20	100	84.47	14.27	35.42	28.53	8.32	7.47	23.34	2.30	1.07	15.33	0.40
S21	104	84.94	11.13	27.75	22.49	6.40	4.40	20.41	2.61	1.07	14.33	0.29
S22	100	84.59	12.83	32.59	27.11	6.88	7.13	23.17	2.34	0.73	13.00	0.42
S23	96	85.53	13.30	34.92	28.80	7.60	8.07	24.25	2.10	0.70	14.33	0.43
S24	96	85.18	13.77	34.00	28.09	7.36	7.20	23.25	2.36	1.00	14.33	0.39
S25	94	84.94	12.73	32.84	26.93	7.28	6.80	23.58	2.16	0.73	15.67	0.43
S26	94	85.41	13.50	32.67	26.31	7.68	6.47	24.42	2.35	0.77	17.67	0.41
S27	98	85.41	13.43	33.84	27.37	7.84	6.80	24.08	2.43	0.87	16.33	0.40
S28	94	85.65	13.37	33.67	27.03	8.00	6.67	25.33	2.46	1.30	19.67	0.43
S29	106	84.71	14.70	33.59	27.73	7.28	6.80	24.67	2.27	1.20	16.67	0.38
S30	100	85.53	11.13	29.25	23.56	6.88	4.07	20.17	2.57	0.93	16.33	0.24

The data obtained of SI are presented in Table 7. The overall Water Quality Index was calculated by adding together each sub index values of each water samples as follows;

$$\mathrm{WQI}=\;\sum {\mathrm{SI}}_{i-n}$$

**Table 7:** Data of sub-index (SI).

**N° sample**	**Tur.**	**pH**	**TDS**	**TH**	**Ca**	**Mg**	**SO**_**4**_^**2−**^	**Alk**	**Cl**^**−**^	**NO**_**3**_^**−**^	**PO**_**4**_^**3−**^	**Cl**_**2**_	**WQI**
S1	13.66	8.52	1.19	1.67	1.99	0.41	0.87	1.71	0.16	0.13	0.63	0.03	30.96
S2	13.41	8.47	1.20	1.71	2.04	0.42	0.82	1.83	0.16	0.14	0.65	0.04	30.89
S3	12.44	8.26	0.93	1.69	2.04	0.40	0.75	1.70	0.15	0.12	0.70	0.05	29.22
S4	14.88	8.23	1.18	1.90	2.26	0.47	0.90	1.78	0.15	0.09	0.72	0.05	32.61
S5	14.39	8.56	1.03	1.76	2.19	0.37	0.79	1.76	0.15	0.09	0.72	0.03	31.84
S6	13.90	8.33	1.08	1.84	2.26	0.41	0.77	2.00	0.15	0.11	0.68	0.04	31.60
S7	12.44	8.29	1.11	1.72	2.09	0.40	0.73	1.92	0.15	0.12	1.02	0.03	30.02
S8	12.20	8.28	1.11	1.80	2.19	0.42	0.74	2.07	0.17	0.12	0.84	0.05	29.98
S9	14.39	8.26	0.95	1.68	2.06	0.38	0.68	1.83	0.16	0.09	0.68	0.03	31.20
S10	11.46	8.26	0.97	1.70	2.03	0.42	0.70	1.88	0.17	0.10	0.72	0.04	28.45
S11	10.98	8.23	1.04	1.74	2.07	0.43	0.62	1.87	0.16	0.13	0.73	0.04	28.03
S12	11.95	8.29	0.98	1.74	2.06	0.44	0.64	1.95	0.14	0.11	0.60	0.05	28.96
S13	10.73	8.22	0.86	1.37	1.64	0.34	0.47	1.48	0.21	0.16	0.59	0.03	26.10
S14	13.17	8.33	1.05	1.73	2.15	0.37	0.81	1.74	0.15	0.14	0.59	0.04	30.27
S15	13.41	8.44	1.06	1.78	2.13	0.43	0.80	1.79	0.16	0.12	0.73	0.04	30.91
S16	13.41	8.22	1.10	1.76	2.12	0.41	0.85	1.68	0.15	0.09	2.31	0.05	32.14
S17	12.93	8.26	1.04	1.73	2.09	0.41	0.78	1.70	0.17	0.11	0.80	0.05	30.08
S18	13.17	8.29	1.03	1.77	2.14	0.41	0.75	1.83	0.16	0.11	0.70	0.05	30.41
S19	12.20	8.30	1.01	1.83	2.17	0.45	0.77	1.74	0.16	0.15	0.76	0.04	29.58
S20	12.20	8.24	1.04	1.73	2.09	0.41	0.73	1.71	0.17	0.13	0.75	0.05	29.23
S21	12.68	8.29	0.81	1.35	1.65	0.31	0.43	1.49	0.19	0.13	0.70	0.04	28.07
S22	12.20	8.25	0.94	1.59	1.98	0.34	0.70	1.70	0.17	0.09	0.63	0.05	28.63
S23	11.71	8.34	0.97	1.70	2.11	0.37	0.79	1.77	0.15	0.09	0.70	0.05	28.76
S24	11.71	8.31	1.01	1.66	2.06	0.36	0.70	1.70	0.17	0.12	0.70	0.05	28.54
S25	11.46	8.29	0.93	1.60	1.97	0.36	0.66	1.73	0.16	0.09	0.76	0.05	28.06
S26	11.46	8.33	0.99	1.59	1.92	0.37	0.63	1.79	0.17	0.09	0.86	0.05	28.27
S27	11.95	8.33	0.98	1.65	2.00	0.38	0.66	1.76	0.18	0.11	0.80	0.05	28.86
S28	11.46	8.36	0.98	1.64	1.98	0.39	0.65	1.85	0.18	0.16	0.96	0.05	28.66
S29	12.93	8.26	1.08	1.64	2.03	0.36	0.66	1.80	0.17	0.15	0.81	0.05	29.93
S30	12.20	8.34	0.81	1.43	1.72	0.34	0.40	1.48	0.19	0.11	0.80	0.03	27.84

The WQI values presented in the last column of Table 7 were compared with the standard values of Table 8. The WQI values at the 30 sampling sites clearly indicate that drinking water in all areas of Azogues is safe to drink. Therefore, based on the general results, drinking water in Azogues is of excellent quality.

**Table 8:** Standard WQI values for water to human consumption.

**WQI range**	**Type of water**	**Explanation**
< 50	Excellent Water	Good for human health
50.1–100	Good Water	Fit for human consumption
100.1–200	Poor Water	Water not in good condition
200.1–300	Very Poor Water	Need attention before use
> 300.1	Inappropriate	Need too much attention
